# Supplementary material for: Pyrolyzed and unpyrolyzed residues enhance maize yield under varying rates of application and fertilization regimes
Source: PeerJ. 2024 Jun 14;12:e17513. doi: 10.7717/peerj.17513 (PMC11182025; doi:10.7717/peerj.17513)
Supplement: Supplemental Information 1 [file peerj-12-17513-s001.docx]

**
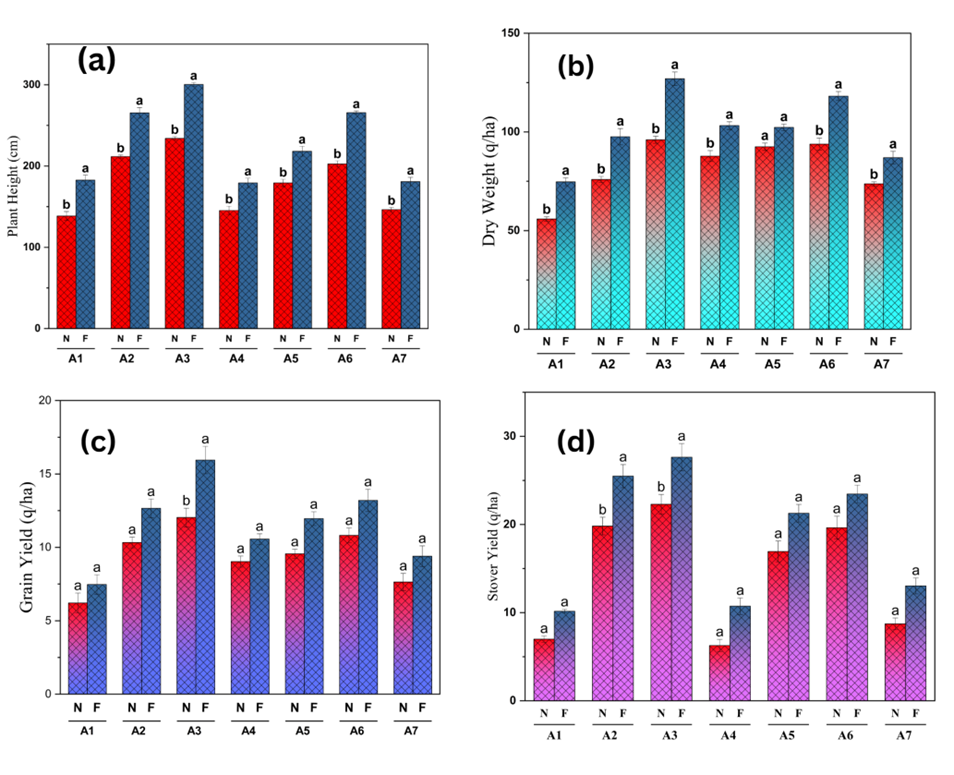
**

**Supp Figure 1 Maize growth and productivity as affected by amendment type and fertilizer regimes.**

**
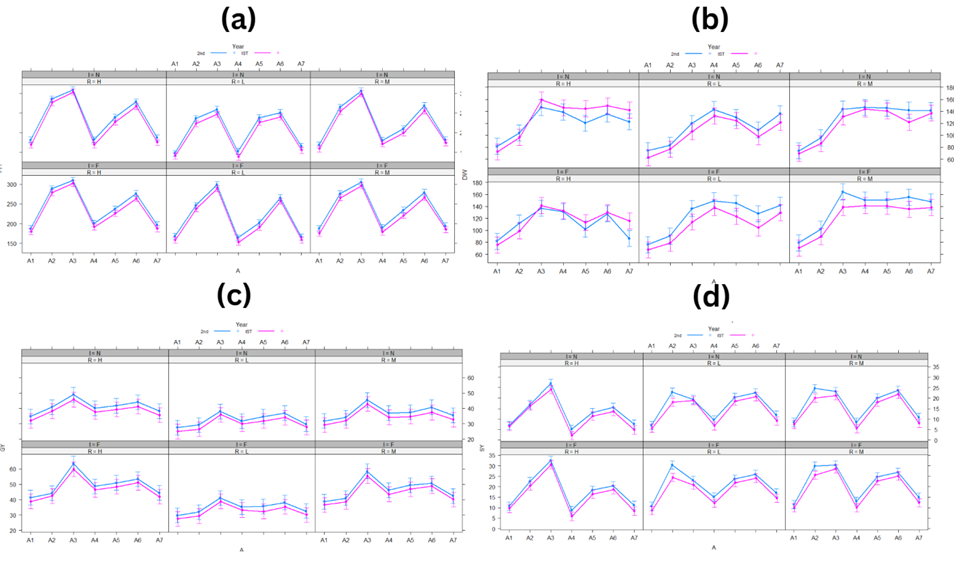
**

**Suppl Figure 2** **Interactive effect of maize growth and productivity on amendment, year, rate of application, and fertilizer regimes.**

**Suppl Table 1 Treatment details**

| **Factor 1 (Amendments)** | **Factor 2 (Rate)** | **Factor 3 (Inorganic fertilizer)** |
| --- | --- | --- |
| 1. No material - (A_1_) | Low - (L) | No fertilizer - (N) |
| 1. Apple biochar (400℃) - (A_2_) | Medium - (M) | RDF (recommended dose of fertilizer) - (F) |
| 1. Apple biochar (600℃) - (A_3_) | High - (H) |  |
| 1. Apple residue biomass - (A_4_) |  |  |
| 1. Dal weed biochar (400℃) - (A_5_) |  |  |
| 1. Dal weed biochar (600℃) - (A_6_) |  |  |
| 1. Dal weed residue biomass - (A_7_) |  |  |
